# Understanding barriers to pediatric hydrocephalus management: an international survey

**DOI:** 10.1007/s00381-026-07273-1

**Published:** 2026-04-22

**Authors:** S. Edwin Ojiako, Rya Muller, Linder H. Wendt, Patrick P. Ten Eyck, Arnold Bhebhe, S. Hassan A. Akbari, Luis Rodriguez, George I. Jallo, Humphrey Kunda, Kirill V. Nourski, Kachinga Sichizya, Christopher M. Bonfield, Rebecca A. Reynolds

**Affiliations:** 1 Department of Neurosurgery, The University of Iowa, Iowa City, IA USA; 2 Iowa Neuroscience Institute, University of Iowa, Iowa City, IA USA; 3 Feinberg School of Medicine, Northwestern University, Chicago, IL USA; 4 Institute for Clinical and Translational Science, University of Iowa, Iowa City, IA USA; 5 Division of Neurosurgery, University Teaching Hospital, Lusaka, Zambia; 6 Division of Pediatric Neurosurgery, Johns Hopkins All Children’s Hospital, St. Petersburg, FL USA; 7 Department of Neurosurgery, Vanderbilt University Medical Center, Nashville, TN USA

**Keywords:** Ventriculoperitoneal shunt, Endoscopic third ventriculostomy, Low- and middle-income countries, High-income countries

## Abstract

**Purpose:**

A pediatric neurosurgeon’s approach to the treatment of hydrocephalus can vary but often includes ventriculoperitoneal (VP) shunt insertion or endoscopic third ventriculostomy (ETV). However, there are several suspected barriers to accessible hydrocephalus care, particularly regarding endoscopy. The aim of this study was to characterize neurosurgical approaches and perceived barriers to pediatric hydrocephalus treatment globally.

**Methods:**

An online survey targeting a non-random sample of pediatric neurosurgeons was distributed via international neurosurgery mailing lists. Demographics including age, sex, level of training, years of practice, and country of residence were collected. Likert-scale and multiple-choice questions were used to determine surgical indications, surgeon preferences, surgeon training, and access to endoscopy. Fisher’s exact test was used to compare frequencies between high- and low-income countries.

**Results:**

Forty-five neurosurgeons from 29 countries with a mean age of 49.2 ± 10.4 (mean ± S.D.) years, all treating pediatric hydrocephalus, responded to the survey. Overall, 64.4% (*n* = 29/45) were from high-income countries (HICs) and 35.6% (*n* = 16/45) were from low- and middle-income countries (LMICs). Regarding VP shunt insertion, 41.4% of HIC respondents and 81.3% (*p* = 0.013) of LMIC respondents acknowledged barriers to treatment. Barriers to VP shunt insertion included equipment access, specifically hardware availability reported by 10.3% of HIC respondents and 43.8% of LMIC respondents (*p* = 0.021). Regarding ETV, 51.7% of HIC surgeons and 93.8% of LMIC surgeons (*p* = 0.007) reported a barrier. Barriers to ETV included equipment access, specifically endoscope/balloon catheter availability, reported by 34.5% of HIC respondents and 81.3% of LMIC respondents (*p* = 0.005). Respondents also reported surgeon/staff training problems, patient financial insecurity, and operating room (OR) access but no significant differences were found between the two economic groups for ETV or VP shunt insertion. Furthermore, 81.3% of LMIC respondents and 44.8% (*p* = 0.027) of HIC respondents expressed the desire to treat more patients endoscopically.

**Conclusion:**

LMICs face more perceived barriers to the treatment of pediatric hydrocephalus than HICs. These perceived barriers are more frequent in endoscopic treatment, in comparison to VP shunt insertion in both economic groups, largely due to improper access to endoscopic equipment. Future studies should focus on leveraging neurosurgical collaborations to improve care worldwide.

**Supplementary Information:**

The online version contains supplementary material available at https://doi.org/10.1007/s00381-026-07273-1.

## Introduction

The higher incidence of hydrocephalus in low- and middle-income countries (LMICs) compared to high-income countries (HICs) may be partly attributed to the high incidence of post-infectious hydrocephalus in LMICs [1]. Hydrocephalus is considered a surgical disease without viable medical treatment options currently available [2, 3].

Philosophies to the surgical treatment of pediatric hydrocephalus vary across the globe [4–7]. Ventriculoperitoneal (VP) shunt and endoscopic third ventriculostomy (ETV) are the most frequently utilized treatment options with newer frontiers currently being explored in certain patient populations such as endoscopic ventricular lavage [2, 8]. A VP shunt is a bellwether procedure in most pediatric neurosurgical practices with ETV gaining traction in certain cases of obstructive hydrocephalus [2, 9]. Choroid plexus cauterization (CPC) has been added to ETV, which has improved the success of the procedure in some hydrocephalus patient populations, specifically infants or patients with communicating hydrocephalus [10]. The selection of a surgical treatment to pediatric hydrocephalus is a nuanced decision with many factors for a neurosurgeon to consider such as age of the patient, hydrocephalus etiology, past surgical history, and available resources [11].

Historically, VP shunt was often considered a “safer” treatment option than ETV due to the likelihood of a successful early outcome [11]. ETV has become more frequently used after landmark studies demonstrated ETV’s safety and efficacy [5, 11]. Since publication, several multinational efforts have augmented ETV availability worldwide through training programs and resource allocation [12, 13]. Surgical approach for hydrocephalus treatment is likely to be surgeon-dependent, patient-dependent, hospital-region-dependent, and resource-dependent [2]. This manuscript seeks to profile the neurosurgical perspective of surgical hydrocephalus treatment and associated perceived treatment barriers that neurosurgeons face in their practices around the world, stratified by country income level. The authors hypothesize that perceived barriers to endoscopic equipment access, surgical training, and medical indigency would be disproportionately evident in LMIC neurosurgical practices.

## Methods

### Study population

From June 2023 until May 2025, an anonymous Google Forms survey [14] targeting a non-random sample of neurosurgeons treating pediatric hydrocephalus was distributed via pediatric neurosurgery list-servs to solicit participation. Specific list-servs included the Congress of Surgeons of East, Central, and Southern Africa, Neurosurgery Research, and author contacts. Respondent emails were checked for duplication and deidentified from the responses to ensure no duplicate responses. A complete list of survey questions is included in Supplementary Table 1. Participants were included if they were actively practicing neurosurgeons who treated children under 21 years of age. Approximately 200 neurosurgeons were contacted from multiple countries in all four World Bank income classifications. These included high-income, upper-middle-income, lower-middle-income, and low-income countries. Geographic regions were also reported descriptively based on World Bank regional designations. These included East Asia and Pacific, Europe and Central Asia, Latin America and the Caribbean, Middle East and North Africa, North America, South Asia, and Sub-Saharan Africa [15].

For analysis, surgeons from both high-income and upper-middle-income countries were collectively categorized as being from HICs, and surgeons from low-income and lower-middle-income countries were collectively categorized as being from LMICs. Although anonymous, to minimize duplicates, survey completions by participants were timestamped and the “limit to 1 response” feature was activated in the settings of the Google Forms survey [14] prompting respondents to sign in prior to participating. Fifty respondents completed the survey (25.0% response rate). Five responses were excluded due to the following: not treating pediatric neurosurgical patients (*n* = 3), not actively treating patients (*n* = 1), and no active neurosurgery department (*n* = 1). Thus, data from 45 respondents were included in the study.

### Ethics

Ethical approval was obtained from the Institutional Review Board at the University of Iowa (IRB-01–202302005). Participants were informed about the level of confidentiality of the study, and consent was obtained at the beginning of the survey via email. All information was collected anonymously, and participants consented to participate in the survey.

### Variables

Variables collected included demographics (age, sex, length/level of training, years of practice, country of residence, type of practice), VP shunt insertion and ETV case volume, treatment indications, types of endoscopic training, and barriers to surgical treatment. Specific perceived barriers queried included operating room (OR) access (time), generic OR equipment availability (drapes and sutures), specific equipment access (shunts, endoscopes, or balloon catheters), patient financial insecurity, and surgeons/staff training. A 5-point Likert scale (1, strongly disagree, to 5, strongly agree) was administered to assess surgical indications, use of the ETV success score [11], training, and the desire to treat more patients endoscopically.

### Statistical approach

Counts and percentages were reported for categorical variables, while means and standard deviations were reported for continuous variables. Likert-scale questions were collapsed into two groups. Those who indicated “Strongly Agree” or “Agree” were grouped together and compared to the frequency of responses “Neither Agree nor Disagree,” “Disagree,” or “Strongly Disagree.” Fisher’s exact tests were used to compare categorical variables between country income categories. The threshold for statistical significance was set a priori at *p* ≤ 0.05, and adjustments for multiple comparisons were not employed due to the exploratory nature of the analysis. Microsoft Excel® and R version 4.1.1 (2024-06−14) were used for all statistical tests.

## Results

### Demographics

Respondents’ demographics and countries are listed in Tables 1 and 2, respectively. A total of 45 respondents from 29 countries were included in the analysis. The average age of respondents was 49.2 ± 10.4 (mean ± SD) years, and 82.2% were male (*n* = 37/45). Most respondents were from HICs (64.4%, *n* = 29/45) and practiced at a public or government hospital (73.3%, *n* = 33/45). Most subjects reported practicing for greater than 10 years (71.1%, *n* = 32/45).

**Table 1:** Demographics

Demographic category	Overall (*n* = 45)	LMIC (*n* = 16)	HIC (*n* = 29)
Average age in years (± SD)	49.2 ± 10.4)	47.9 ± 11.7	49.9 ± 9.8
Male	82.2% (37/45)	81.3% (13/16)	82.8% (24/29)
Level of training			
Practicing neurosurgeon (> 10 years)	71.1% (32/45)	62.5% (10/16)	75.9% (22/29)
Practicing neurosurgeon (1–10 years)	24.4% (10/45)	25% (4/16)	24.1% (7/29)
Resident or registrar	4.4% (3/45)	12.5% (2/16)	0%
Surgeons’ region of residence			
East Asia and Pacific	11.1% (5/45)	25% (4/16)	3.4% (1/29)
Europe and Central Asia	26.7% (12/45)	0%	41.4% (12/29)
Latin America and the Caribbean	13.3% (6/45)	0%	20.7% (6/29)
Middle East and North Africa	11.1% (5/45)	6.3% (1/16)	13.8% (4/29)
North America	13.3% (6/45)	0%	20.7% (6/29)
South Asia	11.1% (5/45)	31.3% (5/16)	0%
Sub-Saharan Africa	3.3% (6/45)	37.5% (6/16)	0%
Completion of a neurosurgical residency training program	91.1% (41/45)	75% (12/16)	100% (29/29)
Average length in years of completed neurosurgical training programs (years) (± SD)	6.0 ± 1.5	4.9 ± 1.4	6.2 ± 1.4
Surgeons’ current type of practice			
Public or government hospital	73.3% (33/45)	81.3% (13/16)	70% (20/29)
Private hospital	15.6% (7/45)	12.5% (2/16)	17.2% (5/29)
University hospital/academic medical center	6.7% (3/45)	0%	10.3% (3/29)
Mission hospital	4.4% (2/45)	6.3% (1/16)	3.4% (1/29)

**Table 2:** List of countries with corresponding number of respondents

Respondents’ country of practice	World Bank income classification	World Bank income region	Number of respondents
Austria	HIC	Europe and Central Asia	1
Brazil	HIC	Latin America and the Caribbean	1
Costa Rica	HIC	Latin America and the Caribbean	1
Croatia	HIC	Europe and Central Asia	1
Denmark	HIC	Europe and Central Asia	1
El Salvador	HIC	Latin America and the Caribbean	1
Greece	HIC	Europe and Central Asia	2
Israel	HIC	Middle East and North Africa	1
Mexico	HIC	Latin America and the Caribbean	3
Poland	HIC	Europe and Central Asia	1
Russia	HIC	Europe and Central Asia	1
Saudi Arabia	HIC	Middle East and North Africa	2
Spain	HIC	Europe and Central Asia	2
Sweden	HIC	Europe and Central Asia	1
Taiwan	HIC	East Asia and Pacific	1
Turkey	HIC	Europe and Central Asia	1
United Arab Emirates	HIC	Middle East and North Africa	1
United Kingdom	HIC	Europe and Central Asia	1
United States	HIC	North America	6
Ethiopia	LMIC	Sub-Saharan Africa	1
India	LMIC	South Asia	3
Mali	LMIC	Sub-Saharan Africa	1
Morocco	LMIC	Middle East and North Africa	1
Nepal	LMIC	South Asia	1
Nigeria	LMIC	Sub-Saharan Africa	2
Pakistan	LMIC	South Asia	1
Sudan	LMIC	Sub-Saharan Africa	1
Vietnam	LMIC	East Asia and Pacific	4
Zambia	LMIC	Sub-Saharan Africa	1
**Total**			**45**

### Training

Most participants from both LMICs 75.0% (*n* = 12/16) and HICs 86.2% (*n* = 25/29) reported receiving dedicated endoscopic training for the treatment of hydrocephalus, though these were not significantly different (*p* = 0.43). The most reported format of training for both groups was live patients at 62.5% (*n* = 10/16) among LMIC participants and 82.8% (*n* = 24/29) among HIC participants (*p* = 0.16). Upon evaluating the perception of training sufficiency, we found no significant differences for VP shunt insertion or ETV, as 81.3% (*n* = 13/16) of LMIC respondents indicated sufficient training in VP shunt insertion, compared to 96.6% (*n* = 28/29) of HIC respondents (*p* = 0.12). Perception of training sufficiency is shown in Table 3. Similarly, 68.8% (*n* = 11/16) of LMIC respondents agreed to having received sufficient training in ETV, which was not significantly different compared to 89.7% (*n* = 26/29) of HIC respondents (*p* = 0.11).

**Table 3:** Dedicated endoscopic training, format of endoscopic training, perception of training sufficiency, use of ETV Success Score and etiological indications for the endoscopic treatment of pediatric hydrocephalus

	Overall (*n *= 45)	LMIC (*n*= 16)	HIC (*n*= 29)	*p *value
Dedicated endoscopic training	82.2% (37/45)	75% (12/16)	86.2% (25/29)	0.43
Format of training of endoscopic training				
Live patients	75.6% (34/45)	62.5% (10/16)	82.8% (24/29)	0.16
Simulation models	46.7% (21/45)	43.8% (7/16)	48.3% (14/29)	> 0.99
Cadaver	44.4% (20/45)	25.0% (4/16)	55.2% (16/29)	0.066
Virtual reality	28.9% (13/45)	37.5% (6/16)	24.1% (7/29)	0.49
Perception of training sufficiency				
ETV	82.2% (37/45)	68.8% (11/16)	89.7% (26/29)	0.11
VP Shunt insertion	91.1% (41/45)	81.3% (13/16)	96.6% (28/29)	0.12
Use of ETV success score	64.4% (29/45)	43.8% (7/16)	75.9% (22/29)	0.051
Etiological indications				
Aqueductal stenosis	80.0% (36/45)	68.8% (11/16)	86.2% (25/29)	0.24
Tectal tumor	66.7% (30/45)	56.3% (9/16)	72.4% (21/29)	0.33
Non-tectal tumor	53.3% (24/45)	43.8% (7/16)	58.6% (17/29)	0.37
Myelomeningocele	37.8% (17/45)	56.3% (9/16)	27.6% (8/29)	0.11
Post-infectious	22.2% (10/45)	31.3% (5/16)	17.2% (5/29)	0.46
IVH	22.2% (10/45)	12.5% (2/16)	27.6% (8/29)	0.29
Other	11.1% (5/45)	12.5% (2/16)	10.3% (3/29)	> 0.99
Desire to treat more patients endoscopically	57.8% (26/45)	81.3% (13/16)	44.4% (13/29)	**0.027**

### Surgical indications

LMIC respondents had a significantly higher desire to treat more patients endoscopically compared to HIC respondents (81.3%, *n* = 13/16 versus 44.4%, *n* = 13/29 respectively, *p* = 0.027). There were no statistically significant differences between the economic groups regarding surgical indications (*p* > 0.05). The reported indications for ETV procedures are shown in Table 3. We found a significant difference in the use of the ETV success score [11] to guide surgical management between LMIC and HIC respondents (43.8%, *n* = 7/16 versus 75.9%, *n* = 22/29 respectively, *p* = 0.051). The use of the ETV success score [11] is shown in Table 3.

### Barriers to hydrocephalus care

Surgeon-reported barriers to treating hydrocephalus (by ETV or VP shunt insertion) are shown in Table 4. Overall, 33.3% (*n* = 15/45) of all respondents reported some form of barrier to pediatric hydrocephalus care regardless of procedure type. With respect to VP shunt insertion, most surgeons reported at least one barrier to treatment (55.6%, *n* = 25/45). The most common barriers reported were as follows: OR access problems (24.4%, *n* = 11/45), hardware availability (22.2%, *n* = 10/45), and patient financial insecurity (20.0%, *n* = 9/45). When stratified by country income group, LMIC respondents reported having at least one barrier to care more frequently than HIC respondents (81.3%, *n* = 13/16 versus 41.4%, 12/29 respectively; *p* = 0.013). The most significant barrier driving this difference was hardware availability, with more surgeons in LMICs citing difficulty (43.8%, *n* = 7/16) than HIC surgeons (10.3%, *n* = 3/29; *p* = 0.021). This was followed by patient financial insecurity, with more LMIC surgeons citing difficulty (37.5%, *n* = 6/16) than HIC surgeons (10.3%, *n* = 3/29; *p* = 0.050). No significant differences were found in surgeons/staff training (*p* > 0.99) or difficulty in OR access (*p* = 0.72) between the two groups.

**Table 4:** Barriers to VP shunt insertion and barriers to ETV

	Overall (*n *= 45)	LMIC (*n *= 16)	HIC (*n *= 29)	*p *value
No overall barriers to care reported	33.3% (15/45)	6.3% (1/16)	48.3% (14/29)	**0.007**
VP Shunt insertion barriers				
No VP shunt insertion barriers to care reported	44.4% (20/45)	18.8% (3/16)	58.6% (17/29)	**0.013**
Hardware availability	22.2% (10/45)	43.8% (7/16)	10.3% (3/29)	**0.021**
Surgeons/staff training issue	2.2% (1/45)	0% (0/16)	3.4% (1/29)	> 0.99
OR access	24.4% (11/45)	18.8% (3/16)	27.6% (8/29)	0.72
Patient financial insecurity	20.0% (9/45)	37.5% (6/16)	10.3% (3/29)	0.050
ETV barriers				
No ETV barriers to care reported	33.3% (15/45)	6.3% (1/16)	48.3% (14/29)	**0.007**
Endoscope/balloon catheter issue	51.1% (23/45)	81.3% (13/16)	34.5% (10/29)	**0.005**
Surgeons/staff training issue	37.8% (17/45)	43.8% (7/16)	34.5% (10/29)	0.75
OR access	20.0% (9/45)	25.0% (4/16)	17.2% (5/29)	0.70
Patient financial insecurity	13.3% (6/45)	25.0% (4/16)	6.9% (2/29)	0.17

Regarding ETV procedures, most respondents reported at least one barrier to treatment (66.7%, *n* = 30/45). Of those reporting a barrier, access to an endoscope and/or available balloon catheter (51.1%, *n* = 23/45) was the most common, followed by surgeons/staff training issues (37.8%, *n* = 17/45). We found a statistically significant difference in the number of respondents that reported facing barriers at 93.8% (*n* = 15/16) in LMICs versus 51.7% (*n* = 15/29; *p* = 0.007) in HICs. Access to an endoscope and/or balloon catheter availability was an issue for 81.3% of LMIC surgeons (*n* = 13/16) compared to 34.5% of HIC surgeons (*n* = 10/29; *p* = 0.005). No significant differences were found in surgeons/staff training issues (*p* = 0.75), patient financial insecurity (*p* = 0.17), or OR access (*p* = 0.70).

## Discussion

Hydrocephalus is the most common condition evaluated in general pediatric neurosurgical practice [1], yet our findings show that surgeons from North America, Europe, South America, Asia, and Africa face perceived challenges in providing treatment. To our knowledge, this is the first study to characterize current challenges faced by pediatric neurosurgeons across the globe in the treatment of hydrocephalus, stratified by income level. Consistent with the authors’ hypothesis, more perceived barriers to treating hydrocephalus were reported by LMIC neurosurgeons compared to those from HICs, which was evident by the proportion of no perceived barriers reported across the economic groups. However, surgeons in both settings described ongoing challenges to patient care [4, 6, 16]. In HIC neurosurgical practices, the most commonly reported barriers in pediatric hydrocephalus treatment were OR access and equipment access problems, mainly as they pertain to endoscope/balloon catheter availability. Conversely, LMIC neurosurgeons reported difficulties with equipment access, specifically to both available endoscope/balloon catheter and VP shunt hardware for implantation, followed by patient financial difficulty. It is important to note that surgical training and hardware availability can stem from and drive different surgical philosophies and strategies to treating hydrocephalus around the world, which ultimately creates variability in the standards of hydrocephalus care [1, 6, 7, 17–19]. This study’s findings are consistent with the authors’ hypothesis that perceived barriers to the treatment of pediatric hydrocephalus are present in many neurosurgical practices, and neurosurgeons in LMICs face more challenges than those in HICs, specifically with respect to equipment availability and patient financial insecurity [17, 20].

### Challenges present in HICs and LMICs

In HICs, OR access problems were among the highest reported barriers by neurosurgeons. This supports findings by Chaganty and Sharma (2021) at a National Health Service (NHS) teaching hospital in the United Kingdom (UK), who showed that a lack of available time in the OR schedule was a barrier due to late starts and early finishes in neurosurgery departments [21]. To address OR inefficiencies in HICs, surgical departments have engaged in systems optimization [22]. Contrary to current literature, OR access problems were not frequently reported by LMIC neurosurgeons. This may be sampling bias but could also be attributed to improvements from global efforts to augment the anesthesia workforce [23], surgical workforce [23], and medical device donations, which all aim to build surgical capacity in LMICs [24]. Overall, the study findings underscore the need for improved OR scheduling and management across the globe. Interestingly, equipment access problems were reported as major concerns by not only LMIC neurosurgeons [17, 25] but also HIC neurosurgeons. In HICs, this could be due to major surgical supply chain problems, leading to limited availability of single-use balloon catheters [26]. This has not been addressed in current neurosurgical literature; however, solutions do exist regarding the broader topic of medical products such as personal protective equipment. This includes building regional equipment stockpiles, increasing competition among manufacturers, advanced commitments by manufacturers to regional hospitals, and the encouragement of domestic manufacturing, which could all be adopted to address neuroballoon supply chain issues [27]. Consistent with current literature, patient financial insecurity was far more reported by LMIC neurosurgeons compared to HIC neurosurgeons [17, 20]. This could be owed to a number of different factors including the lack of universal health coverage in LMICs [28] and the high out-of-pocket expenses leading to catastrophic expenditure in LMICs [29]. A potential solution for this could be the introduction of publicly funded universal health coverage, particularly government-financed systems [30], as Wagstaff et al. found a negative correlation between the incidence of catastrophic payments and health expenditure channeled through social security funds and governmental agencies opposed to private insurance and non-profit institutions [30].

### Shunt insertion for hydrocephalus

The VP shunt is the mainstay of hydrocephalus treatment [31]. In this study, 100% of neurosurgeons report the use of VP shunts in their practice, which supports the sentiment that certain aspects of the surgical treatment of hydrocephalus are similar regardless of geographic setting. Despite its fundamental role in neurosurgery, more than half of neurosurgeons reported a barrier to using VP shunts in their practice, of whom, the overwhelming majority were from LMICs. When compared to ETV, there were fewer reported barriers to VP shunt insertion [32]. However, if VP shunt-related surgery is one of the most common procedures completed in most pediatric neurosurgical practices [31], then this calls into question why significant reported barriers for surgeons have not been addressed. Shunt hardware availability problems and patient financial insecurity were far more commonly reported in LMICs [32], and this is compounded by the high re-operative rates, due to frequent shunt failures [33]. Some countries have reported difficulty in procuring shunt hardware due to the dependence on product donations by non-profit philanthropic organizations as a result of lack of investment in the products by certain hospitals [17]. In some resource-limited countries, the costs of Western-manufactured shunts often exceed the yearly gross national income per capita [15, 34]. In the year 2000, the average cost of a VP shunt implantation in the USA was reported to be $35,816 USD [35]. As such, making these surgeries and implants more affordable is paramount. Cheaper options are available on the market. A clinical trial by Warf et al. reported no significant differences in outcomes between the Codman-Hakim micro-precision shunt with antibiotic-impregnated catheters (estimated 650 USD) and the Indian-manufactured non-antibiotic-impregnated Chabbra shunt (estimated 35 USD) [36]. However, subsequent evidence from a 2019 clinical trial by Mallucci et al. showed that the use of antibiotic-impregnated shunts reduces the risk of infection compared to the use of standard or silver-impregnated shunts and reportedly saves the UK’s NHS £135,753 GBP per infection avoided; thus, further studies are needed [37]. Reliable surgical access to VP shunts should be considered the ideal model of practice for pediatric neurosurgeons and patients worldwide. In contrast, in HICs, the high rate of no perceived barriers being reported to VP shunt insertion was likely due to sufficient access to shunt systems, whereas the reports of perceived barriers to hardware availability likely reflect specific limitations in accessing certain types of shunts at higher institution costs or specific shunt components.

### Endoscopic treatment of hydrocephalus

Choosing a surgical approach to treat pediatric hydrocephalus will vary by surgeon and practice setting and is ultimately guided by knowledge base and resource availability [1, 6, 7, 17–19]. Endoscopic treatment of pediatric hydrocephalus has increased over recent years [38]. This is likely due to the robust efforts of surgical training programs [39] and evidence-based research that has resulted in key clinical tools such as the ETV success score [11]. Many LMIC neurosurgeons under-utilize the ETV success score [11]; however, LMIC study respondents showed a greater desire to utilize ETV in their clinical practice compared to their HIC counterparts. Consistent with reports [40], most respondents in this study reported receiving some endoscopic training, and inadequate endoscope access was the most frequently cited barrier (51.1%, *n* = 23/45). This differs from a 2018 study by Dewan et al., who found that surgeons from lower-income countries reported endoscopy training as a more common barrier than endoscope availability when assessing the worldwide neurosurgical workforce [16]. However, it does align with a 2020 study by Karekezi et al., who reported inadequate endoscope access among formally trained neurosurgeons at the World Federation of Neurosurgical Societies Rabat Training Center [25]. Our findings in combination with the aforementioned studies could indicate the varying needs of surgeons from different LMICs, or better yet, this could be a sign of evolution or perhaps progress, as global health organizations have focused on surgical capacity building through training [41]. In addition to the lack of equipment, inadequate sterilization practices, the shortage of sterilization machines and the exorbitant costs of high-level disinfectants such as Cidex Opa should be noted as undermining the use of specialized equipment such as endoscopes in LMICs [24, 42, 43]. A potentially replicable model is the Duke Global PLUS program, having previously reported success in mitigating these challenges at the Mulago Hospital in Kampala, Uganda, through procurement of autoclaves, staff training in proper sterilization practices and the training of local biomedical engineers in the repair and maintenance of sterilizing equipment [24]. Furthermore, alternative disinfectants such as Sencron2 have been shown to have similar efficacy and a lower price per single reprocessing compared to Cidex Opa [44]. Our findings underscore major equipment and equipment maintenance needs in LMICs. The fact that the need for endoscopic surgical training has decreased from where it was over 7 years ago is not to say that a need still does not exist [16]. Rather, it reflects the more pressing problem of shortages of endoscopic equipment [25], potentially overtaking training needs of surgeons as a barrier [25], although credit should be given to the pediatric neurosurgical community, who seek to train one another in new surgical techniques in the name of progress [45]. Partnerships between neurosurgical departments in HICs and LMICs should continue to foster shared resources and training initiatives to improve global pediatric neurosurgical health [24, 45].

### Additional surgical options

There are several hydrocephalus treatment adjuncts in addition to VP shunt and ETV ± CPC that were not assessed in this survey, which include but are not limited to external ventricular drainage (EVD), use of neuronavigation, and advanced endoscopic techniques such as cyst fenestration or neonatal ventricular lavage [2, 46]. EVDs are a temporary alternative to VP shunt insertion and their utility in treatment is practice-dependent [47, 48]. In this present survey, perceived barriers to EVD were not captured, but they would likely be dependent on institutional infrastructure, sterile practices, and nursing staff support. With respect to neuronavigation, its use has been increasing in neurosurgery [46], but it is suspected to be practice and economic-group dependent. Performing neuronavigation-assisted shunt insertion may increase accuracy and reduce complications, but it depends on the patient, hospital equipment access, and surgeon technical expertise [46, 49, 50]. Lastly, endoscopic lavage for post-hemorrhagic hydrocephalus or endoscopic fenestration in cases of loculated hydrocephalus has been studied in different settings [8], yet definitive clinical trials are currently lacking. Future studies can explore these additional treatment approaches to pediatric hydrocephalus.

## Limitations

There are several limitations to this study. First, our non-random small sample size of 45 pediatric neurosurgeons and 25% response rate as a representation of practice patterns on a national or international scale could limit the study’s external validity, especially when stratified by economic groups, as the majority of respondents represented HICs. Acknowledging this fact, we consider our findings to be exploratory rather than a true representation of the barriers faced in the management of pediatric hydrocephalus. Furthermore, our findings represent perceived barriers of neurosurgeons which are based on their own experiences in practice and such may not represent the true nature of practice patterns and available resources in these countries. There may be regional variation present, but this is outside the scope of this paper, which sought to compare surgeons practicing in countries from different World Bank income groups. We tried several tactics to boost response rate that were within the IRB scope of permission leveraging international list-servs with multiple distributions. Second, list-serv constituents are those with relatively easy access to the larger pediatric neurosurgical community, which could introduce selection bias. We used multiple list-servs in efforts to reduce this bias but were limited to email communications given the international nature of the study. Third, again due to survey distribution via neurosurgical list-servs, there is the potential for underrepresentation of participants practicing in resource-limited regions. This may be due to a hindrance in taking part in internet-based surveys due to the limited resources of the tertiary center and this potentially corresponds with the equipment issues seen mostly in LMICs [16]. A fourth point of consideration is that response bias could result from survey questions that ask respondents’ opinions, which are subject to human perception and may not represent the opinions of other neurosurgeons in the respondent’s country of practice. A fifth point to consider was the scope of the study. This study focused on challenges faced by neurosurgeons in treating pediatric hydrocephalus, specifically regarding the VP shunt insertion and the ETV procedures. Barriers to diagnosing hydrocephalus, imaging surveillance access, and lifelong follow-up care for patients were not addressed but would be an appropriate topic of future study [4]. Despite these limitations, this study represents a worthwhile endeavor as it seeks to understand the global picture of the pediatric neurosurgical approach and challenges to hydrocephalus treatment on an international scale.

## Conclusion

This study characterizes the two most common pediatric neurosurgical approaches to hydrocephalus treatment globally: VP shunt and ETV. Surgical treatment strategies vary worldwide and are guided by surgeon philosophy, training, and resource availability. Study results propose that perceived barriers to the treatment of pediatric hydrocephalus exist more in LMICs compared to HICs. Despite the reported financial insecurity seen in candidates for VP shunt insertion in LMICs, far more reported barriers are faced in endoscopic treatment across both economic groups, which is indicative of a larger global burden to implementation for ETV. The primary issue with endoscopic treatment cited is equipment access. Considering most LMIC neurosurgeons express the desire to treat more patients endoscopically, future studies could focus on leveraging neurosurgical partnership and collaboration to improve versatile treatment access for neurosurgeons and patients worldwide.

## Supplementary Information

Below is the link to the electronic supplementary material.

ESM 1 Supplementary Material 1 (DOCX 27.2 KB)

## Data Availability

Datasets which were generated and underwent analysis in this study are not intended to be made publicly available. This is due to confidentiality and institutional policies. However, they may be made available from the corresponding author upon request.
