# Supplementary material for: Understanding barriers to pediatric hydrocephalus management: an international survey
Source: Childs Nerv Syst. 2026 Apr 22;42(1):167. doi: 10.1007/s00381-026-07273-1 (PMC13102725; doi:10.1007/s00381-026-07273-1)
Supplement: Supplementary file 1 — Supplementary Material 1 (DOCX 27.2 KB) [file 381_2026_7273_MOESM1_ESM.docx]

**Supplementary Table 1: List of Survey questions**

| Question Number | Survey Questions | Answer Options |
| --- | --- | --- |
| 1 | Before continuing, please select your choice below. Clicking 'Agree' means that: I have read the information above. I voluntarily agree to participate. | Agree / Disagree |
| 2 | Country in which you currently reside | Afghanistan / Albania / Algeria / Angola / Antigua and Barbuda / Argentina / Armenia / Australia / Austria / Azerbaijan / Bahamas / Bahrain / Bangladesh / Barbados / Belarus / Belgium / Belize / Benin / Bhutan / Bolivia / Bosnia and Herzegovina / Botswana / Brazil / Brunei / Bulgaria / Burkina Faso / Burundi / Cambodia / Cameroon / Canada / Cape Verde / Central African Republic / Chad / Chile / China / Colombia / Comoros / Congo / Democratic Republic of the Congo / Costa Rica / Côte d’Ivoire / Croatia / Cuba / Cyprus / Czech Republic / Denmark / Djibouti / Dominica / Dominican Republic / East Timor / Ecuador / Egypt / El Salvador / Equatorial Guinea / Eritrea / Estonia / Eswatini / Ethiopia / Fiji / Finland / France / Gabon / Gambia / Georgia / Germany / Ghana / Greece / Grenada / Guatemala / Guinea-Bissau / Guyana / Haiti / Honduras / Hungary / Iceland / India / Indonesia / Iran / Iraq / Ireland / Israel / Italy / Jamaica / Japan / Jordan / Kazakhstan / Kenya / Kiribati / Kuwait / Kyrgyzstan / Laos / Latvia / Lebanon / Lesotho / Liberia / Libya / Liechtenstein / Lithuania / Luxembourg / Madagascar / Malawi / Malaysia / Maldives / Mali / Malta / Marshall Islands / Mauritania / Mauritius / Mexico / Micronesia / Moldova / Monaco / Mongolia / Montenegro / Morocco / Mozambique / Myanmar / Namibia / Nauru / Nepal / Netherlands / New Zealand / Nicaragua / Niger / Nigeria / North Korea / North Macedonia / Norway / Oman / Pakistan / Palau / Panama / Papua New Guinea / Paraguay / Peru / Philippines / Poland / Portugal / Qatar / Romania / Russia / Rwanda / Saint Kitts and Nevis / Saint Lucia / Saint Vincent and the Grenadines / Samoa / San Marino / São Tomé and Príncipe / Saudi Arabia / Senegal / Serbia / Seychelles / Sierra Leone / Singapore / Slovakia / Slovenia / Solomon Islands / Somalia / South Africa / South Korea / South Sudan / Spain / Sri Lanka / Sudan / Suriname / Sweden / Switzerland / Syria / Taiwan / Tajikistan / Tanzania / Thailand / Togo / Tonga / Trinidad and Tobago / Tunisia / Turkey / Turkmenistan / Tuvalu / Uganda / Ukraine / United Arab Emirates / United Kingdom / United States / Uruguay / Uzbekistan / Vanuatu / Vatican / Venezuela / Vietnam / Yemen / Zambia / Zimbabwe |
| 3 | How many neurosurgeons are currently practicing in your country? | |
| 4 | What is your age in years? | |
| 5 | What is your sex? | Female / Male / Prefer not to say |
| 6 | What type of provider are you? | General Surgeon / Neurosurgeon / Other |
| 7 | What is your highest level of training/practice? | Medical student / Resident or Registrar / Fellow / Practicing Neurosurgeon (1-10 years) / Practicing Neurosurgeon (>10 years) / Retired neurosurgeon / Other |
| 8 | Did you complete a neurosurgical residency training program? | Yes / No |
| 9 | If yes, where did you complete your neurosurgical training? | |
| 10 | If yes, how long was your neurosurgical training (in years)? | |
| 11 | Do you treat pediatric patients in your practice? | Yes / No |
| 12 | How many neurosurgeons in your country manage pediatric hydrocephalus? | |
| 13 | Have you received dedicated endoscopic training for treatment of hydrocephalus? | Yes / No |
| 14 | If you have received endoscopic training for treatment of hydrocephalus, what was the format of your training? Check all that apply. | Cadaver, Simulation models, Virtual reality, Live patients, No training received, Other |
| 15 | Hospital at which you currently work | Public or Government hospital / Mission hospital / Private hospital / Other |
| 16 | I frequently treat adult hydrocephalus in my practice | Strongly Disagree / Disagree / Neither agree nor disagree / Agree / Strongly Agree / Not applicable |
| 17 | I frequently treat pediatric hydrocephalus in my practice | Strongly Disagree / Disagree / Neither agree nor disagree / Agree / Strongly Agree / Not applicable |
| 18 | I have received sufficient training on how to place ventriculoperitoneal shunts for hydrocephalus treatment in adults | Strongly Disagree / Disagree / Neither agree nor disagree / Agree / Strongly Agree / Not applicable |
| 19 | I have received sufficient training on how to place ventriculoperitoneal shunts for hydrocephalus treatment in kids | Strongly Disagree / Disagree / Neither agree nor disagree / Agree / Strongly Agree / Not applicable |
| 20 | I have received sufficient training on how to perform endoscopic treatment of hydrocephalus in kids | Strongly Disagree / Disagree / Neither agree nor disagree / Agree / Strongly Agree / Not applicable |
| 21 | I frequently use the "ETV Success Score" by Kulkarni et al in my practice when assessing if patients should receive ETV cs VP shunt for their hydrocephalus (table 1 above) | Strongly Disagree / Disagree / Neither agree nor disagree / Agree / Strongly Agree / Not applicable |
| 22 | I would like to treat more of my patients with hydrocephalus endoscopically than I currently am | Strongly Disagree / Disagree / Neither agree nor disagree / Agree / Strongly Agree / Not applicable |
| 23 | For what etiologies of pediatric hydrocephalus do you more commonly perform ETV compared to VP shunt? (Check all that apply) | Post-infectious, Myelomeningocele, IVH, Aqueductal stenosis, Tectal tumor, Non-tectal brain tumor, None, Other |
| 24 | How many surgeries for new VP shunt insertions for CHILDREN do you estimate are performed annually at your hospital? | |
| 25 | How many surgeries for VP shunt revisions (of existing hardware) for CHILDREN do you estimate are performed annually at your hospital? | |
| 26 | What brand of shunts do you typically implant into patients at your hospital? | Chhabra / Other |
| 27 | If "other" type of shunt is the most common type. Please list it below | |
| 28 | How many ETV operations for CHILDREN do you estimate are performed annually at your hospital? | |
| 29 | How many ETV/CPC operations for CHILDREN do you estimate are performed annually at your hospital? | |
| 30 | What is your driving indication for performing ETV/CPC for a pediatric patient as opposed to only ETV? | |
| 31 | Do you think that endoscopic treatment of hydrocephalus should be performed more often at your facility if safely possible? | Yes / No |
| 32 | Which type of endoscope do you have available at your facility? | Rigid endoscope / Flexible endoscope / Both rigid and flexible endoscope / None |
| 33 | How many endoscopes are available in your country for neurosurgical use? | |
| 34 | What is the brand of the endoscope that you use? (Check all that apply) | Karl Storz, Aesculap, No endoscope available, Other |
| 35 | Where did the endoscope come from that you use for treatment of hydrocephalus? | The hospital purchased it for neurosurgical use. / The hospital purchased for it use by another department (Gynecology, General Surgery, Urology, Orthopedics, or other). / It was donated. / I bought it myself. / I do not use an endoscope. / Other |
| 36 | What are the barriers to the treatment of hydrocephalus at your hospital IN GENERAL? (Check all that apply) | Patient's not presenting for treatment and/or being referred to tertiary center for care, No neurosurgeon who is trained, No available endoscope, No available shunt tubing or valves, No trained OR staff available to assist, No trained nurses available for postoperative care, Lack of available time in the operating theatre, Patient's inability to pay for surgery, No general surgical tools available (suture, drapes, etc), Other |
| 37 | What are the barriers to ENDOSCOPIC treatment of hydrocephalus at your hospital? (Check all that apply) | No surgeon training in endoscopy, No endoscope or associated camera equipment is at my hospital, No balloon catheters for endoscopy, No nursing or OR staff training in endoscope maintenance, I (the surgeon) prefer to place VP shunts, Endoscope is at my hospital, but in disrepair, Endoscope is at my hospital, but is not always available for neurosurgical use since it is shared with other specialties, Endoscope is at my hospital, but no sterilizing capacity (Cidex, autoclave, or other), No general surgical tools available (drapes, suture, etc), Lack of available time in the operating theatre schedule, Patient's inability to pay for the endoscopic procedure, Other |
| 38 | What are the barriers to VP shunt treatment of hydrocephalus at your hospital? (Check all that apply) | Lack of surgeon training in shunting, Lack of available shunt implants (valve or tubing), No surgical tools available (shunt tunneling, suture, drapes, etc.), Lack of available time in the operating theatre schedule, Patient's inability to pay for the VP shunt procedure, Other |
| 39 | Please list your name, current job title, current institution, email address, and country if you would like to be included as a Study Collaborator during publication and results circulation. (Not a required question) | |
| 40 | Any other comments or perspectives that you would like to share about barriers to hydrocephalus treatment at your facility? | |
